# A novel semi-automated classifier of hip osteoarthritis on DXA images shows expected relationships with clinical outcomes in UK Biobank

**DOI:** 10.1093/rheumatology/keab927

**Published:** 2021-12-17

**Authors:** Benjamin G Faber, Raja Ebsim, Fiona R Saunders, Monika Frysz, Claudia Lindner, Jennifer S Gregory, Richard M Aspden, Nicholas C Harvey, George Davey Smith, Timothy Cootes, Jonathan H Tobias

**Affiliations:** Musculoskeletal Research Unit; Medical Research Council Integrative Epidemiology Unit, University of Bristol, Bristol; Division of Informatics, Imaging and Data Science, The University of Manchester, Manchester; Centre for Arthritis and Musculoskeletal Health, University of Aberdeen, Aberdeen; Musculoskeletal Research Unit; Medical Research Council Integrative Epidemiology Unit, University of Bristol, Bristol; Division of Informatics, Imaging and Data Science, The University of Manchester, Manchester; Centre for Arthritis and Musculoskeletal Health, University of Aberdeen, Aberdeen; Centre for Arthritis and Musculoskeletal Health, University of Aberdeen, Aberdeen; Medical Research Council Lifecourse Epidemiology Unit, University of Southampton, Southampton, UK; Medical Research Council Integrative Epidemiology Unit, University of Bristol, Bristol; Division of Informatics, Imaging and Data Science, The University of Manchester, Manchester; Musculoskeletal Research Unit; Medical Research Council Integrative Epidemiology Unit, University of Bristol, Bristol

**Keywords:** OA, dual-energy X-ray absorptiometry, total joint replacement, hip pain

## Abstract

**Objective:**

Conventional scoring methods for radiographic hip OA (rHOA) are subjective and show inconsistent relationships with clinical outcomes. To provide a more objective rHOA scoring method, we aimed to develop a semi-automated classifier based on DXA images and confirm its relationships with clinical outcomes.

**Methods:**

Hip DXAs in UK Biobank (UKB) were marked up for osteophyte area from which acetabular, superior and inferior femoral head osteophyte grades were derived. Joint space narrowing (JSN) grade was obtained automatically from minimum joint space width (mJSW) measures. Clinical outcomes related to rHOA comprised hip pain, hospital diagnosed OA (HES OA) and total hip replacement. Logistic regression and Cox proportional hazard modelling were used to examine associations between overall rHOA grade (0–4; derived from combining osteophyte and JSN grades) and the clinical outcomes.

**Results:**

A toal of 40 340 individuals were included in the study (mean age 63.7), of whom 81.2% had no evidence of rHOA, while 18.8% had grade ≥1 rHOA. Grade ≥1 osteophytes at each location and JSN were associated with hip pain, HES OA and total hip replacement. Associations with all three clinical outcomes increased progressively according to rHOA grade, with grade 4 rHOA and total hip replacement showing the strongest association [57.70 (38.08–87.44)].

**Conclusions:**

Our novel semi-automated tool provides a useful means for classifying rHOA on hip DXAs, given its strong and progressive relationships with clinical outcomes. These findings suggest DXA scanning can be used to classify rHOA in large DXA-based cohort studies supporting further research, with the future potential for population-based screening.

Rheumatology key messages

- Radiographic hip osteoarthritis (rHOA) can be classified semi-automatically on DXA scans.
- rHOA classified in this way showed expected relationships with clinical outcomes related to hip OA.
- DXAs provide a potential means to screen for rHOA and risk of related clinical outcomes.

## Introduction

Hip OA (HOA) is a common condition that is growing in prevalence and leads to 150 total hip replacements (THRs) per 100 000 of population per year in England and Wales [1]. HOA is often classified radiographically (rHOA) based on semi-quantitative scores such as Kellgren–Lawrence (KL) [2] or Croft scoring [3]. Both systems are inherently subjective [4], contributing to widely varying rHOA prevalence estimates that range from 0.9–27% [5], and though atlases help to reduce ambiguity they cannot prevent it entirely [6]. In addition, lower KL and Croft grades are poorly predictive of disease [7], and show weak and inconsistent associations with hip pain, calling into question their clinical relevance [8–10]. This likely reflects not only ambiguity and subjectivity of scoring, but also limitations in how these scores are derived. For example, whereas KL and Croft grading both give equal weighting to joint space narrowing (JSN) and osteophytes, yet where these have been examined individually, osteophyte severity shows a stronger association with hip pain than does JSN [10, 11]. On top of this, when examined in isolation in a large systematic review, minimum joint space width (mJSW), a continuous measure of JSN, showed weak associations with hip symptoms questioning its predominance in these scoring systems [12]. In addition, both grading systems include subchondral sclerosis and cysts despite the lack of evidence that they contribute independently to symptoms [13].

DXA is widely used for diagnosing osteoporosis based on measurements at the spine and hip. Though initially developed for measuring bone mineral density, newer devices have greatly improved resolution, enabling features related to rHOA to be discerned on hip images, such as JSN and osteophytes [14]. Previous small studies have shown DXA-derived hip shape to be predictive of OA progression and total hip replacement (THR), but in these studies the DXA scans were not used to derive rHOA [15]. Due to the low radiation doses involved, DXA is suitable for screening low risk clinical populations, as well as large population-based cohort studies such as UK Biobank, in which ∼40 000 hip DXA scans have been performed to date [16]. Examining hip images in tens of thousands of individuals requires methods that are scalable and ideally automated [17], some of which are now available. Automated calculation of mJSW and digital quantification of osteophyte area are examples of such methods developed on DXAs [11].

The present study was intended to provide a basis for classifying hip DXA scans for rHOA. First, we aimed to semi-automatically annotate and grade JSN and osteophytes in all available UKB participants with hip DXAs. Subsequently, we aimed to categorize the presence of rHOA through the development of a novel classification system giving greater weight to the presence of osteophytes over JSN. Finally, to examine the face validity of our novel grading system, we determined whether UKB participants classified according to rHOA show expected relationships with important clinical OA outcomes, namely prolonged hip pain, hospital diagnosed HOA and subsequent THR.

## Patients & methods

### Population

UKB is a large prospective study that recruited 500 000 adults between 2006–2010. The participants have undergone comprehensive genetic and physical phenotyping (http://biobank.ctsu.ox.ac.uk/crystal/) [18]. This study was approved by UKB (application number 17295) which is overseen by the Ethics Advisory Committee. UKB received ethics approval from the National Information Governance Board for Health and Social Care and North West Multi-centre Research Ethics Committee (11/NW/0382), which covers this study. The UKB extended imaging study has conducted hip DXA scans (iDXA GE-Lunar, Madison, WI, USA) on ∼40 000 individuals to date [16, 19]. All individuals provided informed written consent for this study, which included those UKB participants with a left hip DXA scan available in March 2021. Demographic information was taken from measurements and questionnaires conducted on the same day as the DXA scans.

### DXA-based scoring for hip OA

**Fig. 1:**
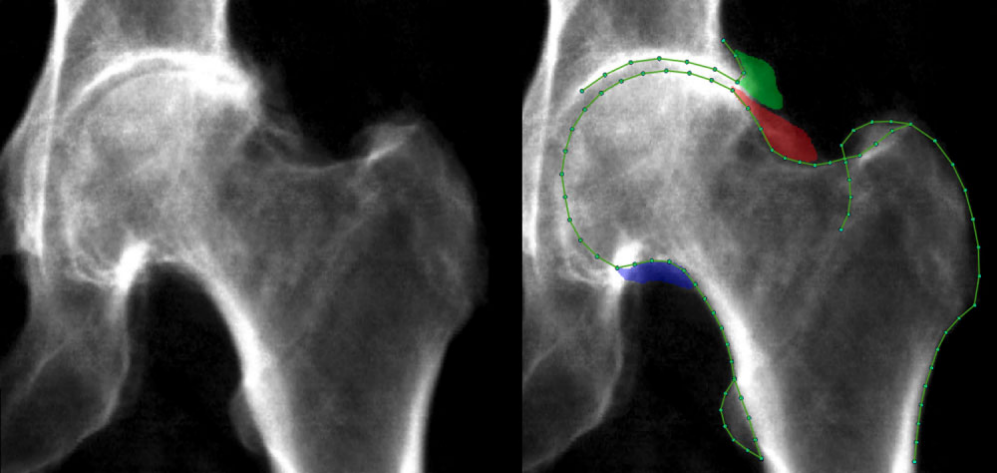
A DXA scan from UK Biobank with features of rHOA Left image is the raw image. Right image is marked with outline points and osteophytes (green: acetabular osteophyte; red: superior femoral head osteophyte; blue: inferior femoral head osteophyte).

A machine learning Random Forest-based algorithm, which was initially trained on ∼7000 manually marked-up images, automatically placed 85 outline points around the left femoral head and acetabulum [11, 20, 21] (Fig.  1). All images were manually checked, which takes less than a min per scan, with 90% of images requiring no point placement correction. Of those images where points required correction, the mean distance of point correction was 1.9 mm. Osteophytes were simultaneously marked up using a custom tool (The University of Manchester) at the lateral acetabulum, superolateral femoral head, and inferomedial femoral head (Fig.  1). Osteophyte grades 1&2 were derived from osteophyte area using previously defined thresholds (grade 1: ≥1 mm^2^, grade 2: ≥10–19 mm^2^ depending on location) [11]; and grade 3 osteophytes were defined as osteophyte area ≥50 mm^2^. Superior minimum joint space width (mJSW) was automatically measured between defined points (Fig.  1) from which JSN grades 1&2 were derived from height-adjusted measures [11]. Additionally, JSN grade 3 was defined as mJSW ≤1.5 mm. Subchondral sclerosis and cysts were not examined due to their relative infrequency [13]. To allow for simple clinical understanding, overall rHOA grade (0–4) was generated using cut-offs, from the sum of osteophyte grades (0–3) at the three locations and JSN grades (0–3), as follows: rHOA grade 0 (sum = 0), grade 1 (sum = 1), grade 2 (sum = 2–3), grade 3 (sum = 4–6), grade 4 (sum = 7–12). These grade classifications were decided after a review of example images and their sum frequencies but prior to the assessment of any associations. The aim was to create grade groupings with visually discernible differences. See Supplementary Methods section 1, available at *Rheumatology* online.

### Clinical outcomes

A binary variable of hip pain persisting for >3months was derived from a questionnaire completed during the participants DXA visit and was not side-specific. Hospital diagnosed HOA was based on international classification of diseases codes released in hospital episode statistics (HES), referred to as HES OA [22]. A total of 400/527 of the included HES OA diagnoses took place after the DXA scan. As there were 127 cases that predate their DXA scan, this variable was examined cross-sectionally. THR was based on Office of Population Censuses and Survey (OPCS) codes. In total, 259/260 THR happened after their DXA scan; the one THR predating the DXA scan was known to be on the right (unimaged) side as the left hip had a native hip imaged and hence THR was examined longitudinally with 259 cases. Neither HES OA nor THR are side-specific. See Supplementary Methods section 2, available at *Rheumatology* online.

### Statistical analysis

Demographic data are shown as mean and range for continuous variables and counts, and frequency for binary variables. Logistic regression was used to examine associations between osteophytes and JSN, and rHOA grades and hip pain and HES OA, results are given as odds ratios (OR) with 95% CI. For ease, we refer to individual features of rHOA such as JSN and osteophytes as endophenotypes of rHOA. When the precise endophenotype and rHOA grade were examined against clinical outcomes, a reference group of those individuals with grade 0 for that exposure was used (i.e. rHOA grades are compared with those with rHOA grade 0). Cox proportional hazard modelling was used to examine associations with THR; results are given as hazard ratios (HR) with 95% CI. The thresholds for semi-quantitative grades of JSN and osteophytes were previously derived in a subsample of 6807 individuals and compared against the same hip pain variable but not HES OA or THR [11]. Therefore, a sensitivity analysis was done excluding these individuals from our hip pain analysis (Supplementary Fig. S1, available at *Rheumatology* online). Directed acyclic graphs informed the *a priori* selection of covariates for the adjusted model, namely age, height, weight and sex. Sex interactions were also examined and sex-stratified analyses presented. Given the sample was 96.8% Caucasian (Supplementary Table S1, available at *Rheumatology* online), ethnicity was not adjusted for. Statistical analysis used Stata version 16 (StataCorp, College Station, TX, USA).

## Results

### Population characteristics

Of the 40 963 available left hip DXAs, 623 were excluded (570 as part of the hip was not visualized, 52 due to poor image quality and one duplicate image) leaving a final sample of 40 340 participants [mean age 63.7 years (range 44–82 years)], comprising 21 046/19 294 (52.2/47.8%) females/males. A total of 3251 (8.1%) reported having had hip pain for >3 months, 527 (1.3%) had a hospital reported diagnosis of HOA (HES OA) and 259 (0.6%) had a THR after their DXA scan (Table  1). The mean duration between DXA scan and THR or study end was 1179 days (range 3–2437) with broadly similar follow-up times between exposure groups.

**Table 1:** Descriptive results

	Males	Females	All
Demographics	Mean [Range]	Mean [Range]	Mean [Range]
Age (years)	64.4 [44–81]	63.0 [45–82]	63.7 [44–82]
Weight (kg)	83.2 [47–171]	68.2 [34–169]	75.4 [34–171]
Height (cm)	177.2 [150–204]	163.6 [135–198]	170.1 [135–204]
*Hip symptoms/outcomes*	Prevalence [%]	Prevalence [%]	Prevalence [%]
Hip pain >3 months	1193 [6.2]	2058 [9.8]	3251 [8.1]
HES OA	220 [1.1]	307 [1.5]	527 [1.3]
THR	106 [0.6]	153 [0.7]	259 [0.6]
Duration from DXA to THR/end of study (mean days [range])	1183 [3–2437]	1174 [3–2436]	1179 [3–2437]
*Ethnicity*	Prevalence [%]	Prevalence [%]	Prevalence [%]
White	18 650 [96.7]	20 396 [96.9]	39 046 [96.8]
Asian	266 [1.4]	171 [0.8]	437 [1.1]
Black	119 [0.6]	134 [0.6]	253 [0.6]
Mixed heritage	61 [0.3]	119 [0.6]	180 [0.5]
Chinese	51 [0.3]	65 [0.3]	116 [0.3]
Unknown	147 [0.8]	161 [0.8]	308 [0.8]
*rHOA measures (grade ≥ 1)*	Prevalence [%]	Prevalence [%]	Prevalence [%]
Any osteophyte (OP)	2570 [13.3]	1443 [6.9]	4013 [10.0]
Acetabular OP	1544 [8.0]	1036 [4.9]	2580 [6.4]
Superior femoral OP	991 [5.1]	502 [2.4]	1493 [3.7]
Inferior femoral OP	810 [4.2]	256 [1.2]	1066 [2.6]
OP at all locations	134 [0.7]	62 [0.3]	196 [0.5]
JSN	2983 [15.5]	1573 [7.5]	4556 [11.3]
*rHOA measures*	Mean [range]	Mean [range]	Mean [range]
Total osteophyte area	24.8 [0.7–438.1]	20.2 [1.4–296.2]	23.2 [0.7–438.1]
Acetabular osteophyte area	16.6 [0.7–200.7]	11.6 [1.4–175.6]	14.6 [0.7–200.7]
Sup femoral osteophyte area	22.2 [2.0–219.9]	23.8 [1.5–140.2]	22.8 [1.5–219.9]
Inf femoral osteophyte area	19.9 [1.7–270.4]	20.2 [1.7–176.1]	20.0 [1.7–270.4]
Minimum JSW	2.97 [0.1–5.9]	2.81 [0.0–5.1]	2.89 [0.0–5.9]
**Total sample**	19 294	21 046	40 340

### Osteophytes and joint space narrowing

Osteophytes were present in 4013 (10%) participants, of which the lateral acetabulum [2580 (6.4%)] was the most common location, followed by the superior femoral head [1493 (3.75%)] and the inferior femoral head [1066 (2.6%)]. Osteophytes were more common in males than females at all locations (Table  1). Osteophytes were larger at the superior femoral head [mean area 22.8 mm^2^ (range 1.5–219.9)], followed by inferior femoral head [mean area 20.0 mm^2^ (range 1.7–270.4)] and acetabulum [mean area 14.6 mm^2^ (0.7–200.7)]. JSN (grade ≥1) was present in 4556 (11.3%) individuals and was more prevalent in males [*n* = 2983 (15.5%)] than females [*n* = 1573 (7.5%)]. Mean mJSW was 2.89 mm (range 0.0–5.9) (Table  1). Prevalence of individual osteophyte and JSN grades are provided in Supplementary Table S2, available at *Rheumatology* online.

#### Osteophytes and joint space narrowing vs clinical outcomes

In analyses adjusted for age, sex, weight and height, osteophytes (grade ≥1) at any site were associated with hip pain, HES OA and THR [OR 2.05 (95% CI 1.85, 2.27), OR 4.98 (4.13, 6.01) and HR 6.17 (4.80, 7.94), respectively] (Table  2). Similar results were seen in unadjusted analyses (Supplementary Table S3, available at *Rheumatology* online). Superior and inferior femoral head osteophytes showed relatively large associations with hip pain [OR 3.04 (2.64, 3.49), 3.45 (2.94, 4.05), respectively], HES OA [OR 8.65 (6.97, 10.73), 8.29 (6.47, 10.60), respectively] and THR [HR 10.31 (7.83, 13.57), 11.76 (8.68, 15.93), respectively] (adjusted analyses). Acetabular osteophytes showed somewhat weaker associations with the clinical outcomes [hip pain: OR 1.83 (1.62, 2.07), HES OA: OR 3.76 (3.02, 4.68), THR: HR 4.30 (3.23, 5.71)]. JSN (grade ≥1) was associated with all three clinical outcomes [hip pain: OR 1.37 (1.23, 1.53), HES OA: OR 3.48 (2.85, 4.23) and THR: HR 3.91 (3.00, 5.09)].

**Table 2:** Adjusted logistic regression results showing the associations between grade ≥1 osteophytes and JSN with hip pain and HES OA

	Hip pain >3months		HES OA		THR	
	OR [95% CI]	*P*	OR [95% CI]	*P*	HR [95% CI]	*P*
Any osteophyte (OP)	2.05 [1.85, 2.27]	2.00 × 10^−43^	4.98 [4.13, 6.01]	1.70 × 10^−63^^*^	6.17 [4.80, 7.94]	1.10 × 10^−45^^*^
Acetabular OP	1.83 [1.62, 2.07]	6.02 × 10^−22^	3.76 [3.02, 4.68]	2.31 × 10^−32^^*^	4.30 [3.23, 5.71]	1.04 × 10^−23^
Superior femoral OP	3.04 [2.64, 3.49]	4.00 × 10^−55^	8.65 [6.97, 10.73]	8.80 × 10^−86^^*^	10.31 [7.83, 13.57]	3.00 × 10^−62^^*^
Inferior femoral OP	3.45 [2.94, 4.05]	2.20 × 10^−52^	8.29 [6.47, 10.6]	2.60 × 10^−63^	11.76 [8.68, 15.93]	5.10 × 10^−57^
OP at all locations	6.95 [5.14, 9.39]	2.51 × 10^−36^	20.53 [14.22, 29.64]	1.60 × 10^−58^	21.79 [14.35, 33.08]	2.10 × 10^−47^
JSN	1.37 [1.23, 1.53]	1.60 × 10^−08^	3.48 [2.85, 4.23]	4.18 × 10^−35^	3.91 [3.00, 5.09]	6.50 × 10^−24^

Adjusted Cox proportional hazard modelling showing the associations between grade ≥1 osteophytes and JSN with THR. Adjusted for age, sex, height and weight.

* denotes a sex interaction term with *P*-value <0.1.

HES OA: hospital diagnosed hip OA; HR: hazard ratio; JSN: joint space narrowing; OR: odds ratio, THR: total hip replacement.

Associations between any, acetabular and superior femoral head osteophyte grade ≥1 and HES OA, and between any superior femoral head osteophyte grade ≥1 and THR showed evidence of a sex interaction (Table  2). In sex-stratified analyses, this appeared to reflect a stronger association in females compared with males, in both unadjusted (Supplementary Tables S4a and S4b, available at *Rheumatology* online) and adjusted (Supplementary Tables S5a and S5b, available at *Rheumatology* online) analyses. For example, in adjusted analyses, HR for the association between superior femoral osteophyte grade ≥ 1 and THR was 7.45 (4.92–11.29) in males compared with 13.32 (9.30–19.09) in females.

**Fig. 2:**
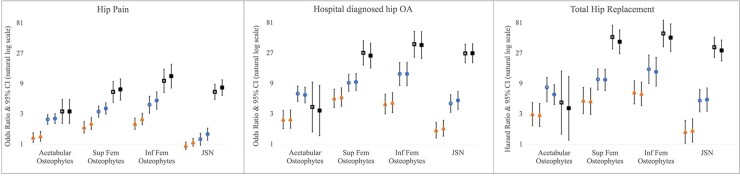
Logistic regression results for the associations between different grades of osteophyte and JSN with hip pain and HES OA Cox proportional hazard modelling results for the associations between grades of osteophyte and JSN with THR. Odds ratios and hazard ratios are plotted with 95% CIs either side comparing each grade of deformity to a reference group of those without that deformity. Results for different clinical outcomes are presented in three different windows. In each graph, triangles represent grade 1 features, circles represent grade 2 features and squares represent grade 3 features. Unadjusted results are shown by hollow shapes and results adjusted for age, height, weight and sex are shown by filled shapes. Y-axis is natural log based.

The associations between individual grades of each endophenotype and hip pain and HES OA were examined using logistic regression, and for THR using Cox proportional hazards modelling, using grade 0 individuals as the reference group. Osteophyte grade was progressively associated with all three clinical outcomes (Fig.  2). JSN grades 1&2 were not associated with hip pain and were only weakly associated with HES OA and THR, whereas a strong association was seen for JSN grade 3 (Fig.  2). Similar associations were observed when excluding those 6807 individuals used to develop our classifier (Supplementary Fig. S1, available at *Rheumatology* online). Sex-stratified analyses showed broadly similar relationships although osteophytes tended to show greater associations with HES OA and THR in females (Supplementary Fig. S2, available at *Rheumatology* online).

### Overall rHOA grade

**Fig. 3:**
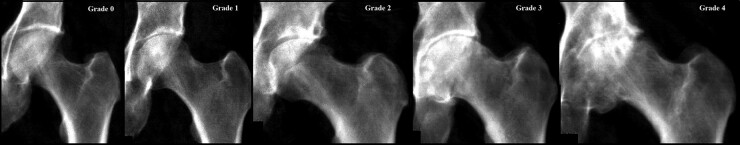
Example UK Biobank DXA scans representing each grade of radiographic hip OA based on the proposed scoring system

Supplementary Table S6 (available at *Rheumatology* online) shows the number of participants per sum of osteophyte and JSN grade (0–12). These sums were used to assign overall rHOA grade: 32 758 (81.2%) of participants had grade 0, 4565 (11.3%) grade 1, 2317 (5.7%) grade 2, 543 (1.3%) grade 3 and 157 (0.4%) grade 4. Each rHOA grade was more common in males, and higher grades were associated with increasing age (Supplementary Table S7, available at *Rheumatology* online). Fig.  3 shows example DXA scans from each rHOA grade.

#### rHOA grade vs clinical outcomes

**Fig. 4:**
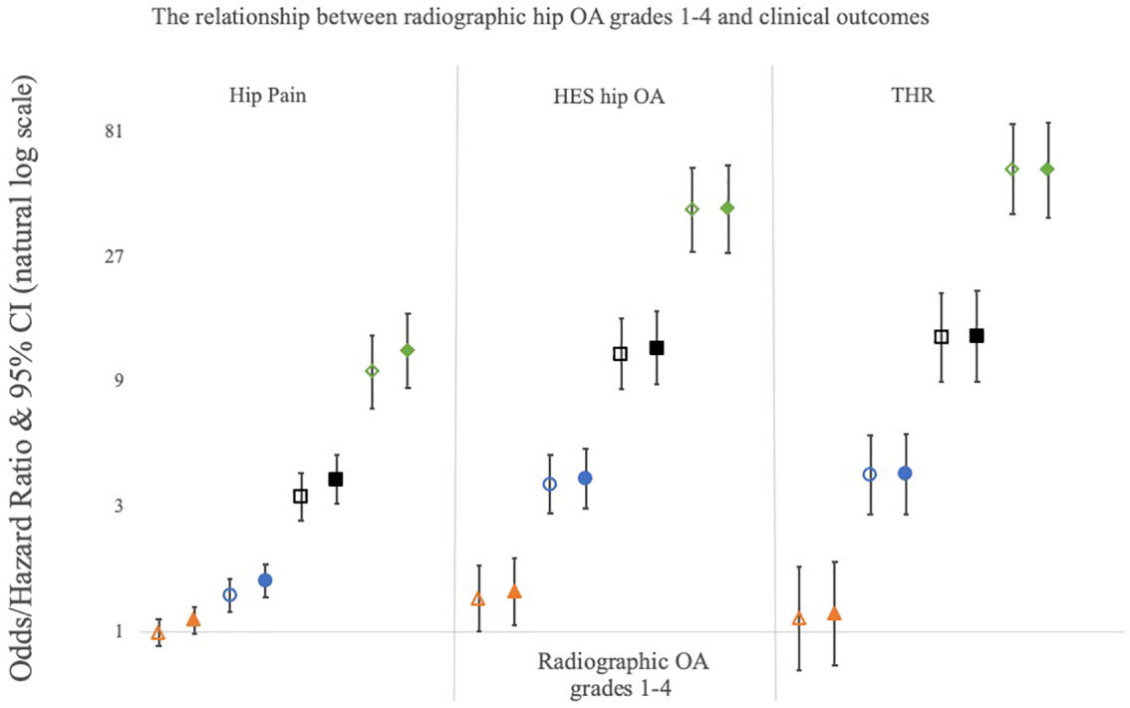
Logistic regression results for the associations between different grades of rHOA and hip pain and HES OA Cox proportional hazard modelling results for the associations between different grades of rHOA and THR. Odds ratios and hazard ratios are plotted with 95% CIs either side comparing each grade to baseline (rHOA grade=0). Results for four different grades of rHOA are presented: triangles represent grade 1, circles represent grade 2, squares represent grade 3 and diamonds represent grade 4. Unadjusted results are shown by hollow shapes and results adjusted for age, height, weight and sex are shown by filled shapes. Y-axis is natural log based.

rHOA grades 1–4 were separately compared with individuals with rHOA grade 0 (*n* = 32 758), in both unadjusted and adjusted logistic regression and Cox proportional hazard models depending on the outcome variable (Fig.  4). There was no or very weak evidence of association between grade 1 rHOA and hip pain, HES OA and THR in both unadjusted and adjusted [OR 1.11 (0.99–1.25), OR 1.42 (1.07–1.90), HR 1.18 (0.75–1.85), respectively] analyses. Grades 2–4 rHOA were associated with hip pain in both unadjusted and adjusted [grade 2: OR 1.57 (1.36–1.81), grade 3: 3.82 (3.08–4.73), grade 4: 11.82 (8.54–16.36)] analyses, with increasing grades showing stronger associations. The same pattern was seen between rHOA grades 2–4 and HES OA in both unadjusted and adjusted [grade 2: OR 3.84 (2.95–5.00), grade 3: 12.08 (8.79–16.61), grade 4: 41.06 (27.94–60.34)] analyses. The strongest associations were seen between rHOA grades 2–4 and THR in both unadjusted and adjusted [grade 2: HR 4.00 (2.80–5.71), grade 3: 13.39 (8.99–19.95), grade 4: 57.70 (38.08–87.44)] analyses. Sex-stratified analyses showed broadly similar relationships between the sexes although females showed stronger relationships with HES OA and THR across all rHOA grades (Supplementary Fig. S3, available at *Rheumatology* online).

## Discussion

We applied semi-automatic methods to annotate and grade osteophytes and JSN on hip DXA scans from 40 340 UKB participants. These were combined using a novel classification system, in which participants were categorized into rHOA grades 0–4. We determined the face validity of these measures by examining their relationships with important clinical OA outcomes, namely prolonged hip pain, HES OA and subsequent THR. Osteophytes, JSN and rHOA showed expected progressive relationships with all three clinical outcomes. For example, participants with the highest grade of rHOA (i.e. grade 4) showed a 58-fold increased risk of subsequent THR.

Our novel DXA-based classification of rHOA has similarities with conventional KL and Croft scoring for OA based on radiographs, in that it divides individuals into five categories based on radiographic features of HOA by increasing severity [2, 3]. In addition, our system of grading osteophytes and JSN is based on Altman and Gold’s atlas [6] that has been widely applied to help standardize the semi-quantitative grading of rHOA [10, 23, 24]. That said, our approach differs in several important ways. Most importantly, our method involves application of machine learning to digital images, enabling automated classification of mJSW, along with a more objective and consistent measurement of osteophytes. A further advantage is that, unlike KL and Croft grading, higher DXA rHOA grades can be achieved in the presence of osteophytes but absence of JSN, which is important given recent findings that osteophytes contribute more to hip pain compared with JSN [11]. In addition, unlike KL and Croft scoring, we did not include subchondral sclerosis or cysts because of their scarcity, neither are well visualized on DXA scans and they both lack evidence that they are independently associated with clinical outcomes [13]. The difficulty visualizing certain characteristics on DXA is also true for medial and inferior JSN, hence we focussed solely on superior JSN.

There are some similarities in comparing our study with previous studies based on KL grading of radiographs. For example, a primary care study (*n* = 1496) found an OR of 17.4 (95% CI 3, 102) for hip pain in those with KL grade 4, compared with an OR of 11.8 (8.5–16.4) for hip pain in those with grade 4 using our DXA-based classification [8]. Previous studies found KL grade >2 to be associated with a HR of 12.9 and OR from 13.8–30.6 for risk of THR, but results were not shown for individual KL grades 3 or 4, which prevents direct comparison with our findings [4, 10, 25]. In the Framingham and OA Initiative studies, where KL or Croft grades were again grouped together, grade >2 on hip radiographs was poorly predictive of hip pain, which led to a shift in clinical guidelines away from routine radiographs for the diagnosis of HOA [7, 26]. The present findings would indicate that, at least using our DXA-based classification system, though less common, higher grades of rHOA show strong associations with hip pain. This finding also fits with the clinical reality that radiographic features of joint degeneration are a pre-requisite for THR [27].

The limited resolution of earlier generations of DXA scanners made it difficult to evaluate radiological features of hip OA [28]. However, a previous study where rHOA was classified by visual inspection of iDXA images concluded that high resolution DXA scanners are a viable option for imaging OA [14]. Whereas DXA-derived hip shape was previously found to be predictive of THR in the Tasmania Older Adult Cohort [15], to our knowledge, this represents the first study where rHOA as measured by DXA was found to be related to a risk of subsequent THR. Understanding the interplay between DXA-derived hip shape and DXA-derived rHOA is beyond the scope of this paper. Further work is warranted to examine if they are independent risk factors for THR or whether they confound/mediate each other’s associations. Furthermore, our findings suggest that, in addition to conventional use for evaluating osteoporosis risk through measurement of BMD, DXA scanners might also have a role in screening for rHOA and the risk of THR, for which they are ideally suited given their low radiation dose, ease of use and widespread availability. Whereas effective disease-modifying drugs for OA (DMOADs) are not yet available, a number of promising lines of discovery are being pursued [29, 30]. If successful, these would provide an incentive for identifying those with rHOA in whom therapy to prevent further progression might be considered.

The prevalence of rHOA depends on its definition and the population [5]. Our study has a mean age of 63.7 years with the youngest participant being 44 years old, meaning it is representative of the general population who are at risk of developing HOA, a condition that tends to present in the later decades of life [31, 32]. The prevalence of rHOA in UKB, defined as grade ≥1, was relatively high at 18.8%. However, 60% of those identified had grade 1 rHOA, which was not associated with hip pain, HES OA or THR, presumably because this group mostly comprised grade 1 JSN [*n* = 2801/4565 (61%)] which we previously found not to be associated with hip pain [11]. Grades 2–4 rHOA were strongly and progressively associated with all three clinical outcomes in this study, largely driven by the presence of osteophytes with 65% of grade 2 rHOA having at least one osteophyte. If rHOA was defined as the presence of rHOA grade ≥2 then 7.5% of UKB participants examined would have rHOA, which is similar to that in previous large cohort studies based on X-rays [4, 5] but lower than others [33, 34], likely reflecting differences in population characteristics such as age. rHOA grade ≥2 was considerably more common in males [*n* = 2086/19 294 (11%)] compared with females [*n* = 931/21 046 (4%)]. This is interesting given previous inconsistent findings on sex differences in rHOA [5, 9, 33, 35] and raises the question of why symptoms and hip replacements are more commonly seen in females despite less degenerative features [1].

We found stronger associations between femoral head osteophytes and clinical outcomes when compared with acetabular osteophytes, which is consistent with previous studies [11, 36]. In particular, one large study using radiographs (*n* = 5839) compared femoral head osteophytes to osteophytes at the femoral head and acetabulum, and their associations with hip pain. In this study, femoral head osteophytes showed stronger associations alone than when combined with acetabular osteophytes [10]. This has possible clinical implications when interpreting hip images as it suggests femoral head osteophytes are most strongly predictive of pain and THR.

The limitations of this study include the clinical outcomes examined being not side-specific, yet we only examine left-sided hip DXAs. However, this would be expected to reduce effect estimates rather than produce spurious associations. DXA scans have inherent disadvantages for evaluating joint morphology and rHOA. For example, medial and inferior aspects of the hip joint are poorly visualized on DXA images, as are certain features related to OA such as sclerosis and bone cysts. In addition, in contrast to radiographs, DXA scans are acquired supine, though the effect of weight bearing on joint space width may be limited [37, 38]. Although our novel scoring system performed well in UKB we have not been able to validate it in an external cohort nor to directly compare it with KL scoring/osteophyte grading on radiographs. Further work is required to confirm its performance. The same is true of our machine learning algorithm that has not been externally validated. Alongside this, UKB is predominantly Caucasian, which means these findings might not be generalizable to different populations.

To conclude, we used semi-automated technology to define osteophyte and JSN grade on high-resolution DXA images, and subsequently combined these to produce an overall rHOA grade based on a novel scoring system giving greater weight to osteophytes. rHOA as determined in this way showed expected associations with clinical features, namely hip pain, HES OA and THR, with higher grades showing greater associations. This provides face validity for using high-resolution DXA scan images to identify rHOA in unselected populations. Taken together, our findings offer new opportunities for using DXA-based cohort studies such as UKB for OA research, and also raise the possibility that DXA scanning may have the potential to screen for OA in unselected patient populations.

## Supplementary Material

keab927_Supplementary_Data

## Data Availability

The data from this study will be available from UK Biobank in a forthcoming data release. Users must be registered with UK Biobank to access their resources (https://bbams.ndph.ox.ac.uk/ams/).
