# Supplementary material for: A novel semi-automated classifier of hip osteoarthritis on DXA images shows expected relationships with clinical outcomes in UK Biobank
Source: Rheumatology (Oxford). 2021 Dec 17;61(9):3586–95. doi: 10.1093/rheumatology/keab927 (PMC9434243; doi:10.1093/rheumatology/keab927)
Supplement: keab927_Supplementary_Data [file keab927_supplementary_data.docx]

Supplementary Methods

1. *DXA mark up, radiographic measures of osteoarthritis*

When osteophytes were marked, they were agreed between two readers BGF (rheumatology doctor) and FRS (postdoctoral researcher), who were trained by an experienced musculoskeletal radiologist MW (see acknowledgments). The training involved the formation and use of a comprehensive DXA-based osteophyte atlas. 500 DXAs selected to include 20% with osteophytes from the first review, were blindly reread for osteophytes >2 months later, giving intrareader kappa’s between 0.80-0.91 for the presence of osteophytes and a concordance correlation coefficient 0.87-0.92 for osteophyte size depending on osteophyte site.

Grade 3 osteophytes were based on an osteophyte area ≥50mm^2^ based on the likelihood of hip pain seen in our previous study (1). JSN grade 3 was defined as mJSW ≤1.5mm based on the previously published likelihood of hip pain and corresponds with other studies conducted on radiographs (2). Osteophyte and JSN quantitative thresholds for grades 1 & 2 were derived, based on Altman and Gold’s atlas, and tested in a subsample of 6807 in UKB against the outcome of hip pain but not HES OA nor THR, as previously published but these individuals are also included in this study (1, 3). To investigate how the rHOA grades performed outside of this development sample (n=6807), a sensitivity analysis was conducted examining the relationships between endophenotype grades and hip pain (relationships with HEA OA and THR were not examined in the previous study) in the 33,533 individuals who did not feature in the previous study (Supplementary Figure 1), the relationships did not differ. For repeatability, automated JSN was reassessed >2months later on 100 images, applying the point placement algorithm and then manually correcting the points where necessary. This gave a kappa of 0.93.

1. *Clinical outcomes*

A binary hip pain variable was derived from the following question: *“Have you had hip pains for more than 3 months?”* The question was asked via questionnaire during the participants DXA visit and was not side-specific. If the question was not answered, it was assumed they did not have hip pain. Hospital diagnosed HOA was based on international classification of diseases (ICD) -9 &10 codes (full list of codes included has previously been published (4)) released in hospital episode statistics (HES) linked to UKB in January 2021. This outcome is referred to as HES OA. HES data has been collected since 1981 in Scotland, 1997 in England and 1998 in Wales. This study examined data that was collected up until 31^st^ December 2020 which is the end point for our Cox proportional hazard models. 400/527 cases of HES OA were diagnosed after their DXA scan in this study, and in these cases the mean duration between DXA and HES OA diagnosis was 2 years. Office of Population Censuses and Survey (OPCS) -3&4 codes are used to record operation procedures in HES, the codes included in this study were OPCS-3: 811, 810, OPCS-4: W371, W81, W391. This data was collected over the same period as described for HES OA and was linked to UKB in January 2021. Although THR was not an exclusion criteria for the extended imaging study, the patient information leaflet stated “you will not be able to take part if you have any metal or electrical implant…in your body”, which meant only one individual had a pre-existing THR in our sample and this was on the contra-lateral side.

1. Patient and Public Involvement

An osteoarthritis focused patient and public involvement group at the University of Bristol (5) reviewed our research plans and supported the overall research aim. The results of this work will be shared with the same group as well as the wider public and patient communities via social media and our university press teams.

|  | Hip pain  Prevalence [%] | HES OA  Prevalence [%] | THR  Prevalence [%] |
| --- | --- | --- | --- |
| White | 3169 [8.1] | 523 [1.3] | 257 [0.7] |
| Asian | 24 [5.5] | 2 [0.5] | 0 [0.0] |
| Black | 21 [8.3] | 0 [0.0] | 0 [0.0] |
| Mixed Race | 12 [6.7] | 0 [0.0] | 1 [0.6] |
| Chinese | 6 [5.2] | 0 [0.0] | 0 [0.0] |
| Unknown | 19 [6.2] | 2 [0.7] | 1 [0.3] |

Supplementary Table S1: The prevalence of clinical outcome measures by ethnic subcategory.

Supplementary Table S2. Count and prevalence figures for grade 1, 2 & 3 endophenotypes of radiographic hip osteoarthritis.

| *rHOA measures* (grade 1) | Males  Prevalence [%] | Females  Prevalence [%] | All  Prevalence [%] |
| --- | --- | --- | --- |
| Any OP | 1452 [3.6] | 908 [2.3] | 2360 [5.9] |
| Acetabular OP | 856 [2.1] | 682 [1.7] | 1538 [3.8] |
| Superior Femoral OP | 530 [1.3] | 254 [0.6] | 784 [1.9] |
| Inferior Femoral OP | 580 [1.4] | 182 [0.5] | 762 [1.9] |
| All OP | 88 [0.2] | 39 [0.1] | 127 [0.3] |
| JSN | 2053 [5.1] | 1257 [3.1] | 3310 [8.2] |
| *rHOA measures* (grade 2) | Prevalence [%] | Prevalence [%] | Prevalence [%] |
| Any OP | 909 [2.3] | 461 [1.1] | 1370 [3.4] |
| Acetabular OP | 585 [1.5] | 336 [0.8] | 921 [2.3] |
| Superior Femoral OP | 389 [1.0] | 199 [0.5] | 588 [1.5] |
| Inferior Femoral OP | 161 [0.4] | 55 [0.1] | 216 [0.5] |
| OP at all locations | 44 [0.1] | 22 [0.1] | 66 [0.2] |
| JSN | 776 [1.9] | 247 [0.1] | 1023 [2.5] |
| *rHOA measures* (grade 3) | Prevalence [%] | Prevalence [%] | Prevalence [%] |
| Any OP | 209 [1.1] | 74 [0.4] | 283 [0.7] |
| Acetabular OP | 103 [0.5] | 18 [0.1] | 121 [0.3] |
| Superior Femoral OP | 72 [0.4] | 49 [0.2] | 121 [0.3] |
| Inferior Femoral OP | 69 [0.4] | 19 [0.1] | 88 [0.2] |
| OP at all locations | 2 [0.01] | 1 [0.0] | 3 [0.01] |
| JSN | 154 [0.8] | 69 [0.3] | 223 [0.6] |

Supplementary Table S3. Unadjusted logistic regression results showing the associations between grade ≥1 osteophytes and JSN with hip pain and HES OA. Unadjusted Cox proportional hazard modelling showing the associations between grade ≥1 osteophytes and JSN with THR. † denotes a sex interaction term with p-value <0.1. CI – confidence interval, HES OA - hospital diagnosed hip osteoarthritis, HR – hazard ratio, JSN – joint space narrowing, OR – odds ratio, THR - total hip replacement.

|  | Hip pain > 3months | | HES OA | | THR | |
| --- | --- | --- | --- | --- | --- | --- |
|  | OR [95% CI] | *P* | OR [95% CI] | *P* | HR [95% CI] | *P* |
| OP any | 1.87 [1.69-2.07] | 6.63 x 10^-35^ | 4.83 [4.02-5.80] | 1.50 x 10^-63 †^ | 6.08 [4.75-7.79] | 1.90 x 10^-46 †^ |
| OP Acet | 1.76 [1.56-1.98] | 9.91 x 10^-20^ | 3.80 [3.06-4.72] | 1.58 x 10^-33 †^ | 4.43 [3.34-5.88] | 5.43 x 10^-25^ |
| OP Sup Fem | 2.68 [2.34-3.07] | 1.80 x 10^-45^ | 8.25 [6.69-10.19] | 6.40 x 10^-86 †^ | 10.04 [7.67-13.15] | 4.40 x 10^-63 †^ |
| OP Inf Fem | 2.92 [2.50-3.41] | 1.10 x 10^-41^ | 7.96 [6.28-10.09] | 1.10 x 10^-65^ | 11.26 [8.42-15.06] | 4.80 x 10^-60^ |
| OP at all locations | 6.17 [4.59-8.30] | 2.35 x 10^-33^ | 20.88 [14.59-29.89] | 6.40 x 10^-62 †^ | 22.86 [15.14-34.53] | 5.00 x 10^-50^ |
| JSN | 1.20 [1.07-1.33] | 1.04 x 10^-03^ | 3.23 [2.67-3.91] | 3.56 x 10^-33^ | 3.75 [2.9-4.85] | 1.06 x 10^-23^ |

Supplementary Table S4a. Male only analyses: unadjusted logistic regression results showing the associations between grade ≥1 osteophytes and JSN with hip pain and HES OA. Unadjusted Cox proportional hazard modelling showing the associations between grade ≥1 osteophytes and JSN with THR. CI – confidence interval, HES OA - hospital diagnosed hip osteoarthritis, HR – hazard ratio, JSN – joint space narrowing, OR – odds ratio, THR - total hip replacement.

| Male | | | | | | |
| --- | --- | --- | --- | --- | --- | --- |
|  | Hip pain > 3months | | HES OA | | THR | |
|  | OR [95% CI] | *P* | OR [95% CI] | *P* | HR [95% CI] | *P* |
| Any osteophyte (OP) | 2.11 [1.83-2.43] | 4.03 x 10^-25^ | 4.12 [3.13-5.43] | 6.46 x 10^-24^ | 5.06 [3.45-7.42] | 1.04 x 10^-16^ |
| Acetabular OP | 1.95 [1.64-2.31] | 5.02 x 10^-14^ | 3.19 [2.30-4.42] | 3.42 x 10^-12^ | 3.63 [2.35-5.63] | 7.40 x 10^-09^ |
| Superior femoral OP | 2.77 [2.30-3.34] | 2.04 x 10^-26^ | 5.82 [4.23-8.02] | 3.75 x 10-^27^ | 7.53 [4.97-11.40] | 1.49 x 10^-21^ |
| Inferior femoral OP | 3.43 [2.82-4.15] | 6.75 x 10^-36^ | 8.30 [6.07-11.33] | 2.50 x 10^-40^ | 10.94 [7.28-16.45] | 1.37 x 10^-30^ |
| OP at all locations | 7.14 [4.93-10.34] | 2.01 x 10^-25^ | 16.63 [10.14-27.29] | 9.18 x 10^-29^ | 19.52 [10.92-34.88] | 1.09 x 10^-23^ |
| JSN | 1.41 [1.22-1.63] | 4.81 x 10^-06^ | 3.58 [2.73-4.71] | 6.18 x 10^-20^ | 4.49 [3.06-6.58] | 1.30 x 10^-14^ |

| Female | | | | | | |
| --- | --- | --- | --- | --- | --- | --- |
|  | Hip pain > 3months | | HES OA | | THR | |
|  | OR [95% CI] | *P* | OR [95% CI] | *P* | HR [95% CI] | *P* |
| Any osteophyte (OP) | 2.05 [1.77-2.37] | 2.45 x 10^-22^ | 6.45 [5.03-8.26] | 5.10 x 10^-49^ | 8.22 [5.94-11.38] | 5.29 x 10^-37^ |
| Acetabular OP | 1.83 [1.54-2.17] | 7.71 x 10^-12^ | 4.81 [3.60-6.44] | 3.72 x 10^-26^ | 5.66 [3.91-8.20] | 4.72 x 10^-20^ |
| Superior femoral OP | 3.34 [2.72-4.10] | 1.51 x 10^-30^ | 13.60 [10.23-18.07] | 2.50 x 10^-72^ | 15.62 [10.95-22.29] | 5.70 x 10^-52^ |
| Inferior femoral OP | 3.63 [2.75-4.79] | 8.31 x 10^-20^ | 10.66 [7.22-15.73] | 9.20 x 10^-33^ | 17.88 [11.65-27.46] | 1.10 x 10^-39^ |
| OP at all locations | 6.74 [4.06-11.18] | 1.57 x 10^-13^ | 34.34 [19.91-59.23] | 4.83 x 10^-37^ | 35.75 [19.82-64.51] | 1.54 x 10^-32^ |
| JSN | 1.24 [1.06-1.46] | 7.84 x 10^-03^ | 3.43 [2.59-4.53] | 4.90 x 10^-18^ | 3.79 [2.62-5.47] | 1.17 x 10^-12^ |

Supplementary Table S4b. Female only analyses: unadjusted logistic regression results showing the associations between grade ≥1 osteophytes and JSN with hip pain and HES OA. Unadjusted Cox proportional hazard modelling showing the associations between grade ≥1 osteophytes and JSN with THR. CI – confidence interval, HES OA - hospital diagnosed hip osteoarthritis, HR – hazard ratio, JSN – joint space narrowing, OR – odds ratio, THR - total hip replacement.

Supplementary Table S5a. Male only analyses: adjusted logistic regression results showing the associations between grade ≥1 osteophytes and JSN with hip pain and HES OA. Adjusted Cox proportional hazard modelling showing the associations between grade ≥1 osteophytes and JSN with THR. Adjusted for age, height and weight. CI – confidence interval, HES OA - hospital diagnosed hip osteoarthritis, HR – hazard ratio, JSN – joint space narrowing, OR – odds ratio, THR - total hip replacement.

| Male | | | | | | |
| --- | --- | --- | --- | --- | --- | --- |
|  | Hip pain > 3months | | HES OA | | THR | |
|  | OR [95% CI] | *P* | OR [95% CI] | *P* | HR [95% CI] | *P* |
| Any osteophyte (OP) | 2.06 [1.78-2.37] | 1.93 x 10^-23^ | 4.01 [3.04-5.28] | 6.41 x 10^-23^ | 4.82 [3.28-7.07] | 9.36 x 10^-16^ |
| Acetabular OP | 1.88 [1.58-2.23] | 1.33 x 10^-12^ | 3.10 [2.23-4.30] | 1.43 x 10^-11^ | 3.46 [2.23-5.37] | 2.81 x 10^-08^ |
| Superior femoral OP | 2.82 [2.33-3.40] | 6.68 x 10^-27^ | 5.88 [4.26-8.11] | 3.34 x 10^-27^ | 7.45 [4.92-11.29] | 2.73 x 10^-21^ |
| Inferior femoral OP | 3.31 [2.73-4.02] | 1.42 x 10^-33^ | 7.80 [5.69-10.67] | 1.38 x 10^-37^ | 10.00 [6.63-15.07] | 3.71 x 10^-28^ |
| OP at all locations | 7.17 [4.94-10.41] | 4.03 x 10^-25^ | 16.31 [9.90-26.88] | 6.34 x 10^-28^ | 18.66 [10.44-33.36] | 5.65 x 10^-23^ |
| JSN | 1.44 [1.24-1.67] | 1.85 x 10^-06^ | 3.62 [2.75-4.78] | 6.96 x 10^-20^ | 4.44 [3.02-6.52] | 3.26 x 10^-14^ |

Supplementary Table S5b. Female only analyses: adjusted logistic regression results showing the associations between grade ≥1 osteophytes and JSN with hip pain and HES OA. Adjusted Cox proportional hazard modelling showing the associations between grade ≥1 osteophytes and JSN with THR. Adjusted for age, height and weight. CI – confidence interval, HES OA - hospital diagnosed hip osteoarthritis, HR – hazard ratio, JSN – joint space narrowing, OR – odds ratio, THR - total hip replacement.

| Female | | | | | | |
| --- | --- | --- | --- | --- | --- | --- |
|  | Hip pain > 3months | | HES OA | | THR | |
|  | OR [95% CI] | *P* | OR [95% CI] | *P* | HR [95% CI] | *P* |
| Any osteophyte (OP) | 2.06 [1.78-2.38] | 3.00 x 10^-22^ | 6.04 [4.70-7.76] | 3.80 x 10^-45^ | 7.36 [5.31-10.20] | 4.63 x 10^-33^ |
| Acetabular OP | 1.81 [1.52-2.15] | 2.57 x 10^-11^ | 4.48 [3.34-6.00] | 1.13 x 10^-23^ | 5.04 [3.48-7.31] | 1.51 x 10^-17^ |
| Superior femoral OP | 3.44 [2.79-4.23] | 2.57 x 10^-31^ | 12.42 [9.30-16.58] | 2.00 x 10^-65^ | 13.32 [9.30-19.09] | 3.50 x 10^-45^ |
| Inferior femoral OP | 3.78 [2.86-5.00] | 1.35 x 10^-20^ | 9.28 [6.25-13.78] | 2.13 x 10^-28^ | 14.33 [9.29-22.12] | 2.64 x 10^-33^ |
| OP at all locations | 6.7 [4.02-11.16] | 3.04 x 10^-13^ | 27.81 [15.95-48.50] | 1.00 x 10^-31^ | 25.69 [14.13-46.72] | 1.93 x 10^-26^ |
| JSN | 1.32 [1.12-1.55] | 8.49 x 10^-04^ | 3.33 [2.50-4.42] | 1.20 x 10^-16^ | 3.45 [2.38-5.01] | 7.84 x 10^-11^ |

Supplementary Table S6. The breakdown of individual rHOA sums.

| rHOA sums | Count [Prevalence] | Cumulative Count [Prevalence] |
| --- | --- | --- |
| 0 | 32758 [81.2] | 32758 [ 81.2] |
| 1 | 4565 [11.3] | 37323 [92.5] |
| 2 | 1813 [4.5] | 39136 [97.0] |
| 3 | 504 [1.3] | 39640 [98.3] |
| 4 | 272 [0.7] | 39912 [98.9] |
| 5 | 155 [0.4] | 40067 [99.3] |
| 6 | 116 [0.3] | 40183 [99.6] |
| 7 | 61 [0.2] | 40244 [99.8] |
| 8 | 43 [0.1] | 40287 [99.9] |
| 9 | 29 [0.1] | 40316 [99.9] |
| 10 | 11 [0.0] | 40327 [100.0] |
| 11 | 11 [0.0] | 40338 [100.0] |
| 12 | 2 [0.0] | 40340 [100.0] |

Supplementary Table S7. Summary of rHOA prevalence across rHOA grades.

| rHOA Grade | Male Count  [Prevalence] | Female Count  [Prevalence] | Combined Count [Prevalence] | Mean age [Range] |
| --- | --- | --- | --- | --- |
| 0 | 14471 [75.0] | 18287 [86.9] | 32758 [81.2] | 63.5 [44-82] |
| 1 | 2737 [14.2] | 1828 [8.7] | 4565 [11.3] | 64.1 [45-81] |
| 2 | 1577 [8.2] | 740 [3.5] | 2317 [5.7] | 64.7 [46-81 |
| 3 | 396 [2.1] | 147 [0.7] | 543 [1.3] | 65.5 [47-80] |
| 4 | 113 [0.6] | 44 [0.2] | 157 [0.4] | 67.1 [49-79] |
| Total | 19294 | 21046 | 40340 |  |


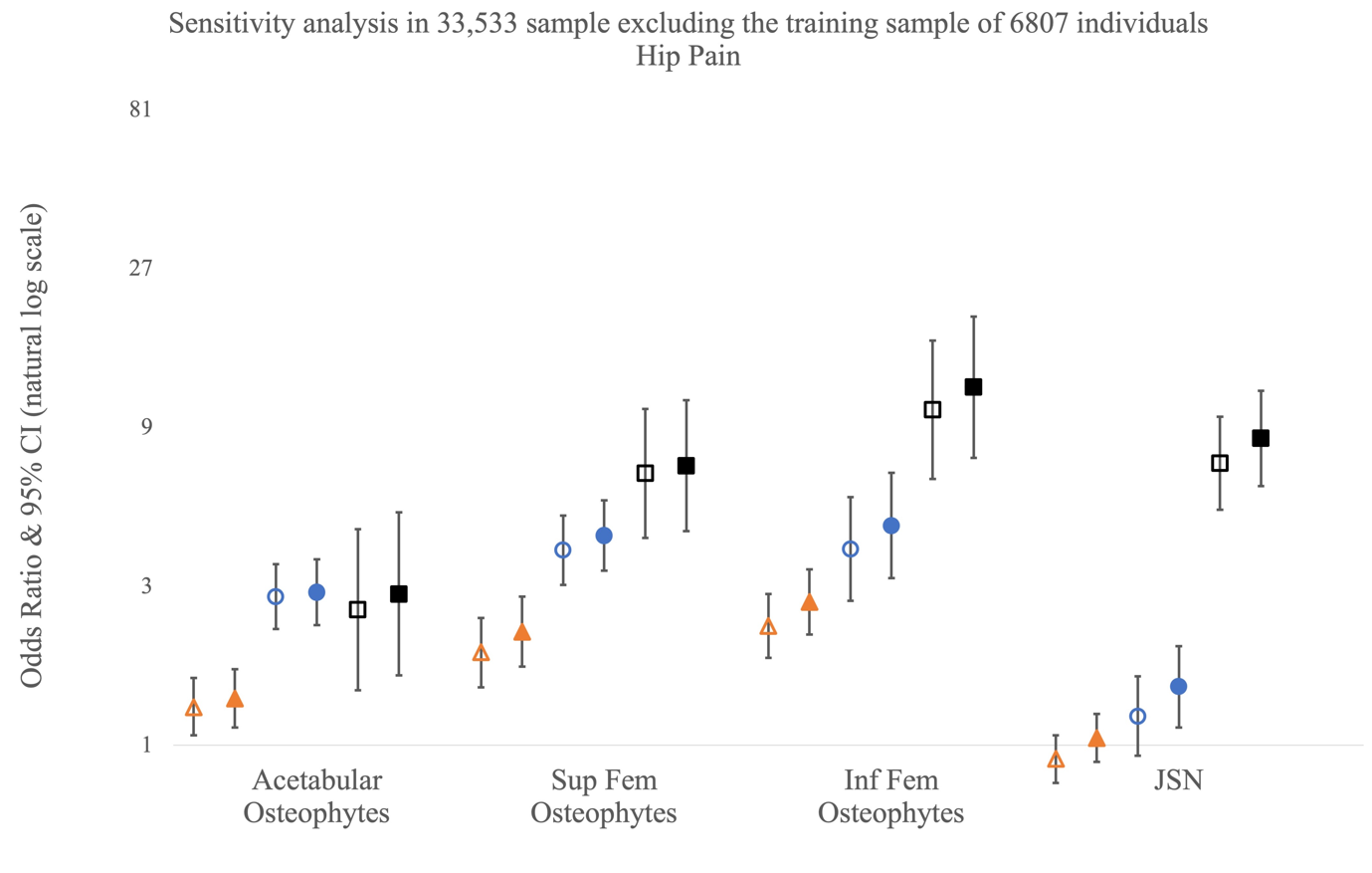
Supplementary Figure S1. Logistic regression results for the associations between different grades of osteophyte and JSN, and hip pain in 33,533 individuals excluding 6,807 individuals that were used to define grades of osteophytes and JSN. Odds ratios are plotted with 95% confidence intervals either side. In each graph, triangles represent grade 1 features, circles represent grade 2 features and squares represent grade 3 features. Unadjusted results are shown by hollow shapes and results adjusted for age, height, weight and sex are shown by filled shapes. Y-axis is natural log based.


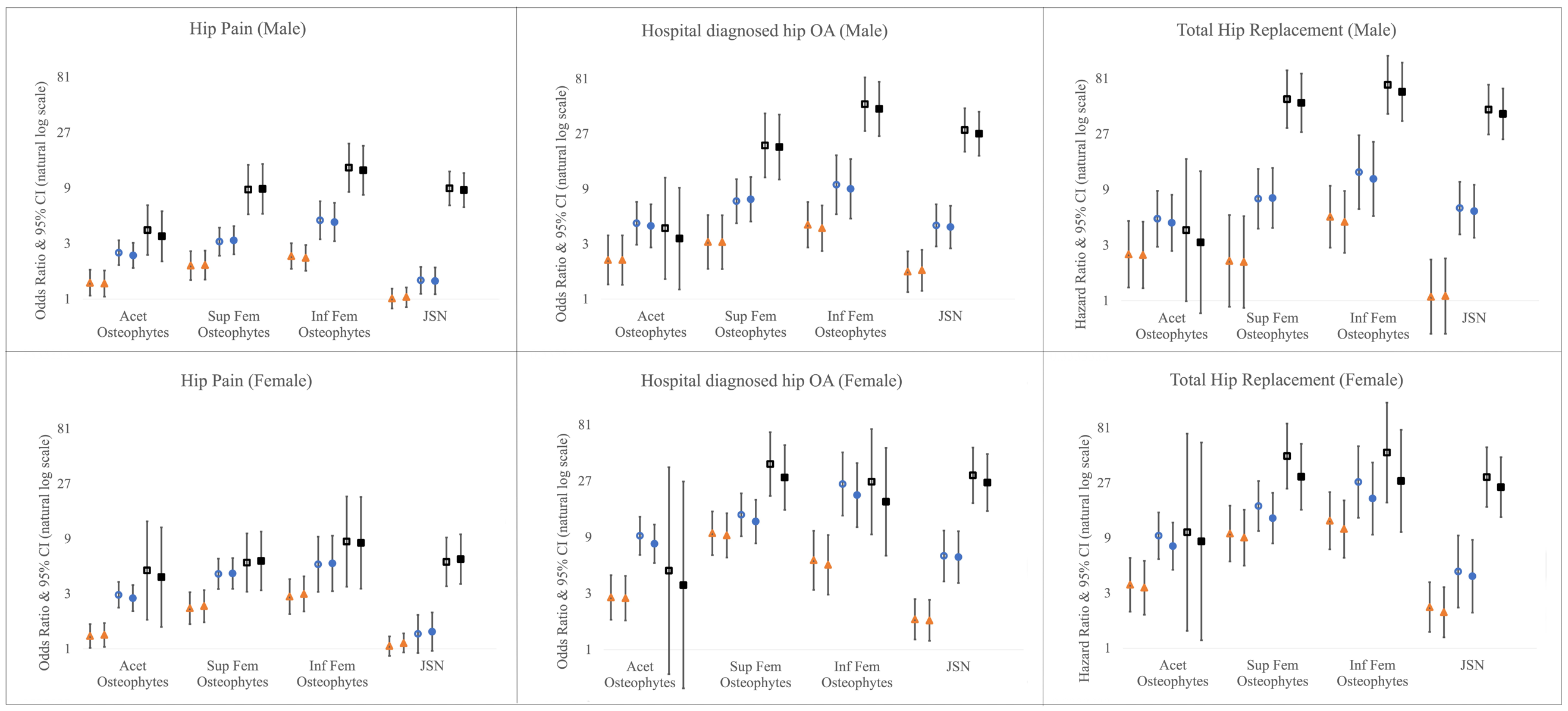


Supplementary Figure S2.

Figure 2. Sex-stratified analyses: logistic regression results for the associations between different grades of osteophyte and JSN with hip pain and HES OA. Cox proportional hazard modelling results for the associations between different grades of osteophyte and JSN with THR. Odds ratios and hazard ratios are plotted with 95% confidence intervals either side. Results for different clinical outcomes are presented in three different windows per sex. In each graph, triangles represent grade 1 features, circles represent grade 2 features and squares represent grade 3 features. Unadjusted results are shown by hollow shapes and results adjusted for age, height and weight are shown by filled shapes. Y-axis is natural log based.


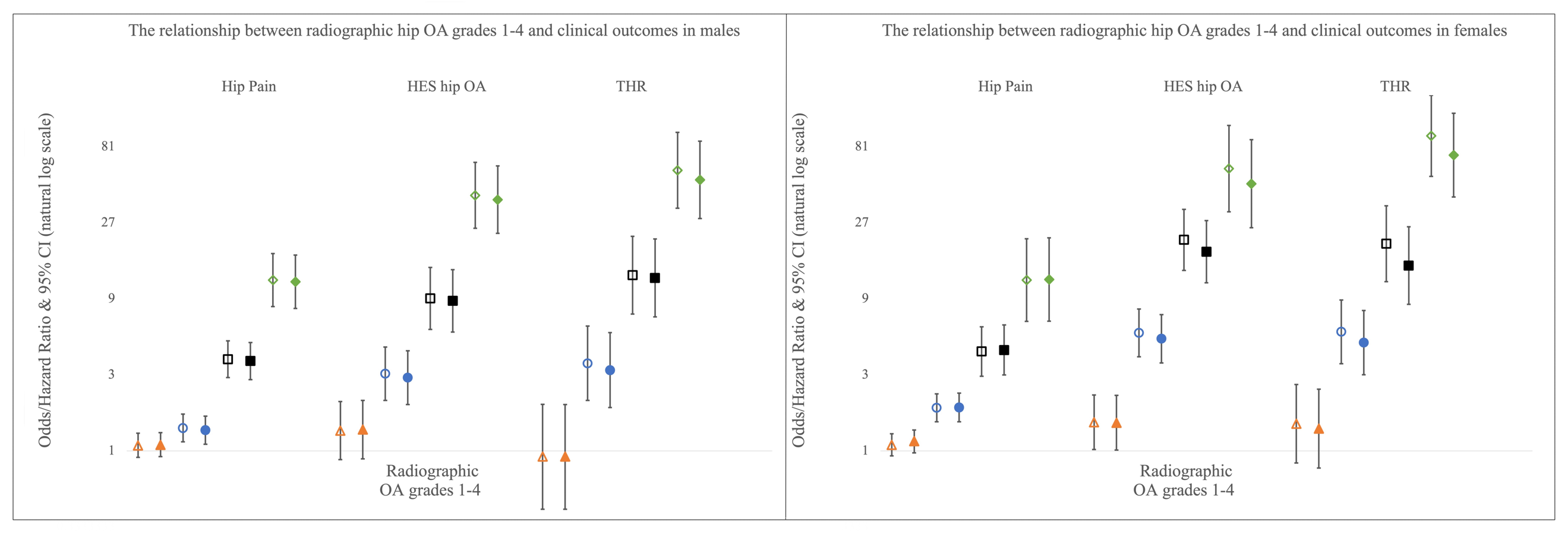


Supplementary Figure S3. Sex-stratified analyses: logistic regression results for the associations between different grades of rHOA with hip pain and HES OA. Cox proportional hazard modelling results for the associations between different grades of rHOA with THR. Odds ratios and hazard ratios are plotted with 95% confidence intervals either side comparing each grade to baseline (rHOA grade=0). Results for four different grades of rHOA are presented, triangles represent grade 1, circles represent grade 2, squares represent grade 3 and diamonds represent grade 4. Unadjusted results are shown by hollow shapes and results adjusted for age, height and weight are shown by filled shapes. Y-axis is natural log based.

References:

1. Faber BG, Ebsim R, Saunders FR, Frysz M, Lindner C, Gregory JS, et al. Osteophyte size and location on hip DXA scans are associated with hip pain: findings from a cross sectional study in UK Biobank. Bone. 2021:116146.

2. Croft P, Cooper C, Wickham C, Coggon D. Defining osteoarthritis of the hip for epidemiologic studies. American Journal of Epidemiology. 1990;132(3):514-22.

3. Altman RD, Gold GE. Atlas of individual radiographic features in osteoarthritis, revised. Osteoarthritis Cartilage. 2007;15 Suppl A:A1-56.

4. Zengini E, Hatzikotoulas K, Tachmazidou I, Steinberg J, Hartwig FP, Southam L, et al. Genome-wide analyses using UK Biobank data provide insights into the genetic architecture of osteoarthritis. Nat Genet. 2018;50(4):549-58.

5. Gooberman-Hill R, Burston A, Clark E, Johnson E, Nolan S, Wells V, et al. Involving patients in research: considering good practice. Musculoskeletal Care. 2013;11(4):187-90.
